# Post-Mortem of a Case of Hodgkin’s Disease

**Published:** 1880-03-20

**Authors:** 


### *DESCRIPTION AND POST-MORTEM OF A CASE OF HODGKIN'S DISEASE.*

The following interesting case of this curious disease is described by Dr. M. Charters in the Medical Press and Circular :

*Previous history.*—The patient, F. R., æt. sixteen, a tinsmith, was admitted into the Royal Infirmary on March 7th, 1879, having been recommended to my care by one of the physicians of Anderson's College Dispensary. His mother stated that about four years ago she first observed a swelling about the size of a marble below the right lobe of the ear. Two years afterwards a similar swelling appeared below the left lobe of the ear. About six months ago she noticed the thyroid gland swollen, and subsequent to this the glands in the armpit and left inguinal region were enlarged. Since these latter swellings began, the patient has been feeling weak, unable to work, and gradually becoming thinner. The other members of his family, four in all, were healthy-

*Condition of admission.*—The patient was observed to be a spare, thin, emaciated lad. His face was pale, and, on undressing, the veins of the chest and abdomen were markedly prominent. The glands below the lobes of both ears were swollen to the size of an egg. The other glands of the neck, and particularly those along the border of the sterno-mastoid, were also enlarged. The thyroid was also enlarged to a considerable extent, and so also were the axillary glands of both sides. It was noteworthy, however, that while the inguinal glands of the left side were increased in size, those of the right remained normal. The glandular swellings were movable, and were neither red, tender, nor painful. The percussion note of the left side of the chest was duller than the right, and crepitation was observed in the left infra-clavicular region. The cardiac sounds were normal.

The patient complained of pain on pressure both in the hepatic and splenic regions, but there was no abnormal dullness either of the liver or spleen. Blood drawn from the finger and placed under the microscope showed no increase of the white corpuscles. The latter fact, and the non-enlargement of the spleen, were, during life, the main elements for diagnosing the case to be not of leucocythæmia, but of lymphadenoma—the Hodgkin's disease of England, the œdema of Trousseau, the pseudo-leucæmia of some authors. It may also be mentioned that the urine was acid, of sp. gr. 1021, and was free then and during all the illness, from albumen.

For the first two days the patient could walk across the room, although his gait was tottering and uncertain. After that he preferred to keep to his bed, and preserved a listless attitude, rarely choosing to answer questions, and preferring simply to be still, and caring little for either food or drink. His temperature, which was carefully taken, indicated 99° or 100° Fahr. in the morning, while in the evening it rose to 103° or 104°. Copious perspirations followed even the slightest sleep. Gradually he became weaker. The difficulty of breathing increased, and he was unable to retain any food. For the last two days of his life he was delirious, and his lips and mouth were covered with sores.

At the morning visit of the 22d of March his face was flushed, the breathing was great, and he appeared as if he was choking. To give temporary relief to his distressing condition, Dr. Cameron performed tracheotomy. Twelve hours afterwards the patient died quietly.

Mr. Nelis' report of the *post mortem* examination is as follows :

Body spare and emaciated. Superficial glands very much enlarged in cervical, axillary and inguinal regions. On opening the chest the mediastinal glands are found to be very much enlarged.

*Lungs.*—The right lung was apparently normal, except at its base, where it has some swollen nodules of white tissue resembling lymphatic glands. In the apex of the left lung there are a number of patches of yellow, softening tubercle, and there is also a cavity about the size of a walnut, which contains a quantity of semi-fluid, whitish material. The rest of the lung tissue is congested.

*Spleen.*—Normal in size, rather pale in color. On section it presents a number of small, angular, whitish-yellow-looking bodies. There is a recent effusion of lymph around the hylus, and along the upper surface; and mixed up with this there is a small amount of blood.

*Liver.*—On section, presents a slightly yellowish, greasy, solid appearance, but is not otherwise abnormal.

*Kidneys* normal. Supra-renal bodies normal. Mesenteric glands everywhere enlarged.—*Half-Yearly Comp. of Med. Science.*

